# Synthesis of Nocistatin C-terminal and it^’^s Amide Derivatives as an Opioid Peptide

**Published:** 2016

**Authors:** Enayatollah Sheikhhosseini, Saeed Balalaie, Mohammadali Bigdeli

**Affiliations:** a *Department of Chemistry, Kerman Branch, Islamic Azad University, Kerman, Iran. *; b *Peptide Chemistry Research Center, K. N. Toosi University of Technology, P. O. Box 15875-4416, Tehran, Iran. *; c *Faculty of Chemistry, Kharazmi Uinversity, No. 49, Mofateh Ave. Tehran, Iran.*

**Keywords:** Solid phase peptide synthesis, Nocistatin, Amidation, C-Terminal amidated peptides

## Abstract

A new biological active hexapeptide of C-terminal of nocistatin, contains Glu-Gln-Lys-Gln-Leu-Gln sequence was synthesized according to solid phase peptide synthesis on the surface of 2-chloro tritylchloride resin and using fmoc-protected amino acids in the presence of TBTU (O-(Benzotriazol-1-yl)-N,N,N',N'-tetramethyl uranium tetrafluoroborate) as a coupling reagent. Then, amidation of the C-terminus of peptides was carried out using NH_4_Cl and alkyl ammonium chloride (RNH_3_Cl) in the presence of TBTU and a tertiary amine (DIPEA) as the base at room temperature in good to high yields. Cleavage of the desired peptides from the surface of the resin after the addition of TFA (1%) provided the protected peptides. All of the products were purified using preparative HPLC and structures were assigned according to MALDI-mass spectrometry data.

## Introduction

Nocistatin is a new biologically active peptide which is produced from the same precursor as nociceptin. Tests have indicated that these two peptides may play opposite roles in pain transmission. Nocistatin blocks nociceptin-induced allodynia and hyperalgesia and attenuates pain evoked by prostaglandin E_2 _(1-3). The hexapeptide of C-terminal of nocistatin (Glu-Gln-Lys-Gln-Leu-Gln), which is conserved in bovine, human, and murine species which possess allodynia-blocking activity. Furthermore, intracheal pretreatment with anti-nocistatin antibody decreases the threshold for nociceptin-induced allodynia. Although nocistatin does not bind to the nociceptin receptor, it binds to the membrane of mouse brain and spinal cord with high affinity. 

Despite several dozen of studies which describe diverse biological actions of nocistatin, only small steps have been taken towards understanding the nature of its receptor. Some research results have provided more strong pieces of evidence that a G protein–coupled receptor for nocistatin exists on neurons and nocistatin inhibits the release of [^3^H]5-HT in a concentration-dependent manner (4). UliZeilhofer *et al.* showed that nocistatin inhibited synaptic glycine and GABA release onto neurons in spinal cord slices. They also demonstrated that the effects of nocistatin were abolished by pertussis toxin and did not involve NOP receptors (5). Finally, it is possible for nocistatin to act as an already identified receptor, but in an unusual manner that is not readily detected in screening assays; for example, by acting as an allosteric enhancer of agonist action (4, 5).

The presence of a C-terminal of amido group on the peptide chain is essential for the biological activity of many peptides. It has been shown that derivatization of the terminal carboxyl function of peptides to get alkylamides could be expected to lead to derivatives that would not only be resistant to the attack of carboxypeptidases, but also possess higher binding affinity for the opioid receptors due to enhanced hydrophobicity at C-terminus (6, 7). It could also affect the lipophilicity of the peptides and could affect interaction with opiate receptors (7, 8). Meanwhile, lengthening and shortening the alkyl chain has been found to have an adverse effect on the antinociceptive activity of peptides (8-10). There are multiple ways for preparing carboxamides and peptides from carboxylic acids and amines, many of which (using reagents such as; BOP, DCC, HOBt, and PyBOP (11, 12), Mukaiyama›s reagent (13) have been used for more than 20 years. PhSiH_3_ (14), LiAlSeH (15), and AlMe_3_-mediated (16) have been used more recently. In general, these agents actin situ as activating reagents and convert the carboxylic acids into more reactive intermediates.

In continuation of our research work to design a novel and efficient approach for the amidation of C-terminal peptides (17) and synthesis of potential tumor imaging agent (18), we wish herein to report an approach to the synthesis of hexapeptide of nocistatin contains Glu-Gln-Lys-Gln-Leu-Gln sequence and it^,^s amide derivatives via solid phase peptide synthesis. 

**Scheme 1:**
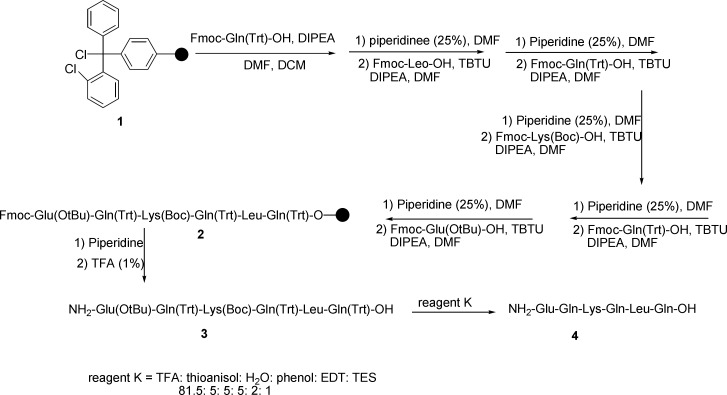
Total synthesis of nocistatin C-terminus peptide

**Scheme 2:**
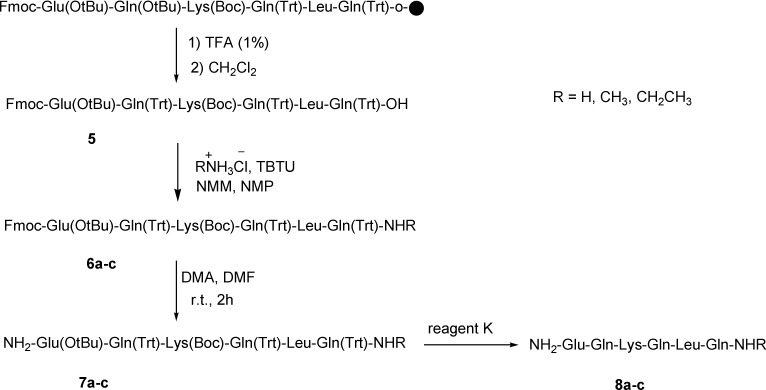
Total synthesis of amidated nocistatin C-terminus peptide

**Figure 1:**
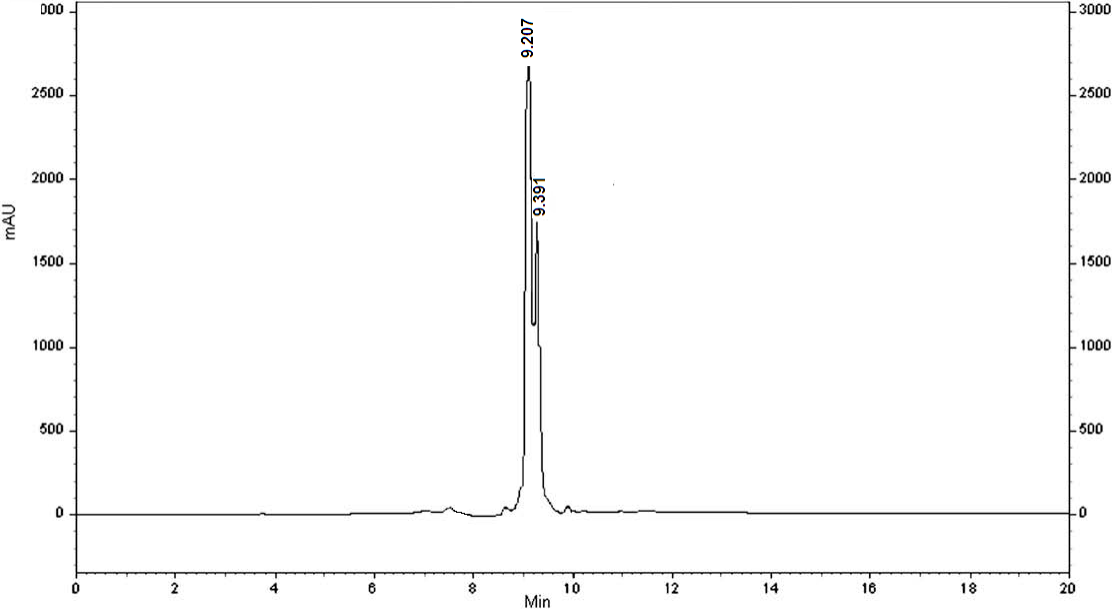
The HPLC profile of nocistatin C-terminal 4

**Figure 2:**
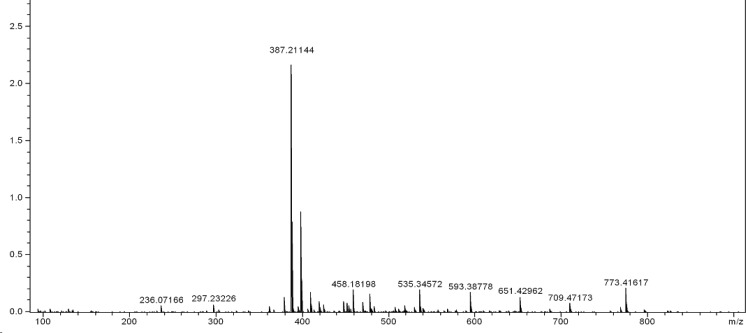
Mass spectrum (ESI) of compound 4

**Figure 3:**
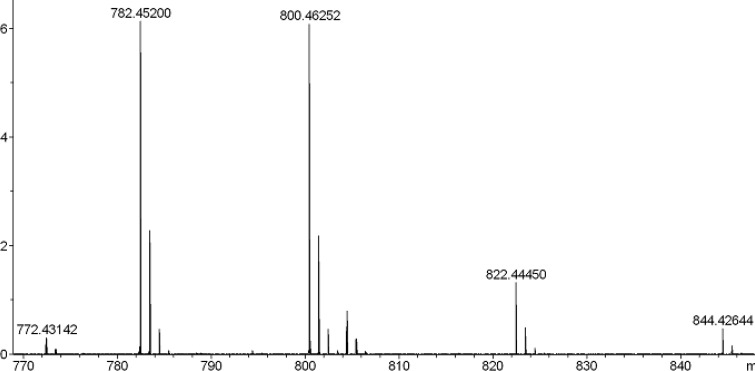
Mass spectrum (ESI) of compound 8c.

## Experimental

*Materials:* Commercially available materials were used without further purification. Reactions were monitored by thin layer chromatography (TLC). Mass spectra were obtained using a *MALDI-MS BrukerApexQe FT-ICR. *Purification was done using Prep-HPLC pump 1800 and Anal-HPLC pump 1000 (KNAUER).

*General procedure for the synthesis of hexapeptide Glu-Gln-Lys-Gln-Leu-Gln:* Synthesis was carried out using 2-chlorotrityl chloride resin (1.0 mmol/g) following standard fmoc strategy.Fmoc-Gln-OH (10 mmol) was attached to the 2-CTC resin (5.0 g) with DIPEA (6.85 mL, 40 mmol) in anhydrous DCM: DMF (50 mL, 1:1) at room temperature for 2 h. After ﬁltration, the remaining tritylchloride groups were capped by a solution of DCM/MeOH/DIPEA (17:2:1, 120 mL) for 30 min. The resin was ﬁltered and washed thoroughly with DCM (1× 20 mL), DMF (4× 20 mL) and MeOH (5× 20 mL). The loading capacity was determined by weight after drying the resin under vacuum and was 0.9. The resin-bound fmoc-amino acid was washed with DMF (3× 20 mL) and treated with 25% piperidine in DMF (65 mL) for 30 min and the resin was washed with DMF (3× 20 mL). Then a solution of fmoc-Leu-OH (7.5 mmol), TBTU (2.40 g, 7.5 mmol), DIPEA (3.0 mL, 17.5 mmol) in 30 mL DMF was added to the resin-bound free amine and shaken for 1 h at room temperature. After completion of coupling, resin was washed with DMF (4× 20 mL) and DCM (1× 20 mL). The coupling was repeated as the same methods for other amino acids of the sequence. In all cases for the presence or absence of free primary amino groups, Kaiser test was used. Fmoc determination was done using UV spectroscopy method. After completion of couplings, resin was washed with DMF (4× 20 mL), DCM (1× 20 mL). The produced hexapeptide 3 was cleaved from resin by treatment of TFA (1%) in DCM (275 mL) and neutralization with pyridine (4%) in MeOH (85 mL). The solvent was removed under reduced pressure and the protected peptide was precipitated after water addition. The yield was 80%. The ﬁnal deprotection of the protected peptide was performed by reacting with reagent K (20 mL/g peptide) for 2 h at room temperature. The ﬁnal peptide 4 was dried under vacuum at 40 ^°^C (yield: 70%).

*General procedure for amidation of C-terminus nocistatin (8a-c).*

To a solution of 0.264 g TBTU (0.82 mmol) and RNH_2_.HCl (1.1 mmol) in 1.5 cm^3^ NMP were added peptide 5 (0.55 mmol) and 0.3 cm^3^ NMM (2.73 mmol). The mixture was stirred overnight. The progress of reaction was monitored using TLC. The C-terminus amidated peptides (6a-c) were precipitated in water and then dried. For fmoc-deprotection of 6a-c, the reaction was stirred in a solution of diethylamine (0.5 mL) in DMF (5 mL) for 2 h at room temperature. The solvent was removed under reduced pressure and diethylether was added, then, precipitated peptides (7a-c) were dried under vacuum at 40 °C. The final deprotection was done using reagent K (TFA: thioanisol: H_2_O: phenol: EDT: TES; 81.5: 5: 5: 5: 2: 1) and in this way all of protecting groups were removed. The final peptides were dried under vacuum at 40 ºC (the yields of isolated products were 70-80%). Further purification was done using Prep-HPLC with column (ODS-C18, 120 × 20 mm) and UV detector (λ = 210 nm). Eluents A (CH_3_CN/water mixture (70/30)) and B (NaH_2_PO_4_/water 10 mM) were used for in a gradient program with a flow of 30 mL/min, 0-10 min: 100% B; 10-15 min: 0-15% A; 10-35 min: 15% A; 35-37 min: 15-100% A; 37-47 min: 100% A; 47-50 min: 100% B; 50-60 min: 100% B. The mass spectral data for the compounds 4 and 8a-c are as follows: HR-MS (ESI) calcd for 4 (C_32_H_57_N_10_O_12_) [M+H] 773.41320, found 773.41617. Calcd for 8a (C_32_H_58_N_11_O_11_) [M+H] 772.43121, found 772.43151; calcd for 8a (C_32_H_57_N_11_NaO_11_) [M+Na] 794.41316, found 794.41352. Calcd for 8b (C_33_H_60_N_11_O_11_) [M+H] 786.44655, found 786.44645; Calcd for 8c (C_34_H_62_N_11_O_11_) [M+H] 800.46248, found 800.46252; calcd for 8c (C_34_H_61_N_11_NaO_11_) [M+Na] 822.44443, found 822.44450.

## Results and discussion

Synthesis of nocistatin C-terminal (Glu-Gln-Lys-Gln-Leu-Gln) was done according to solid phase peptide synthesis on the surface of 2-chlorotritylchloride resin and using fmoc-protected amino acids in the presence of TBTU as a coupling reagent. Then, an efficient route was reported for the amidation and alkylamidation of the C-terminus peptides using ammonium chloride and alkylammonium chloride in the solution phase at room temperature. Details for the synthesis of amide derivatives of nocistatin C-terminus peptide are summarized in Schemes 1 and 2.

Hexapeptide 2 was manually synthesized using the standard solid phase peptide synthesis via Fmoc strategy. First of all, Fmoc-Gln-OH was loaded on the surface of 2-chlorotrityl chloride resin 1 and Fmoc-protected amino acids were coupled according to the known methods until the resin-bound hexapeptide 2 was afforded. Cleavage of the desired peptide from the surface of the resin after the addition of piperidine provided the protected peptide 3. The final deprotection of the protected peptide was performed by reagent K and nocistatin 4 was obtained.

Nocistatin C-terminus peptide could be amidated with ammonium chloride, methylammonium chloride, and ethylammonium chloride and lead to the amidated form of C-terminus peptide 5 (Scheme 2).

This amidation reaction was carried out using TBTU as a coupling reagent and DIPEA as a base at room temperature. The reaction yields were good and details were shown in the experimental section. For full deprotection of the hexapeptide, a mixture of TFA: thioanisol: H_2_O: phenol: EDT: TES (81.5: 5: 5: 2: 1) was used. The synthetic details were clarified in the experimental section. The crude products were of good purity in general, as shown by analytical HPLC analysis (Figure 1.). All the products were characterized using ESI mass spectrometry (Figure 2, 3.).

Molecular weights of all the target compounds were confirmed. The molecular ion peak (M+1)^+^ was identified at m/z = 773.41617 for 4 and 800.46252 for 8c. 
